# Clinicopathological features, therapeutic options, and significance of CD103 expression in 15 patients with follicular mucinosis

**DOI:** 10.3389/fmed.2023.1032072

**Published:** 2023-02-17

**Authors:** Jiaqi Wang, Yanqing Wang, Hongyu Zhou, Ping Wang, Mengyan Zhu, Liuyu Li, Hong Shen

**Affiliations:** ^1^Department of Dermatology, Hangzhou Third People’s Hospital, Affiliated Hangzhou Dermatology Hospital of Zhejiang University School of Medicine, Hangzhou, China; ^2^Department of Dermatology, Hangzhou Third People’s Hospital, Zhejiang Chinese Medical University, Hangzhou, China

**Keywords:** follicular mucinosis, mycosis fungoides, tissue resident memory T cell, CD103, TRM cell

## Abstract

**Background:**

Follicular mucinosis (FM) is generally divided into a primary benign idiopathic form and a secondary form associated with mycosis fungoides.

**Objective:**

To analyze the clinical and pathological features of FM and explore the pathological significance of CD103 expression.

**Methods:**

In this case series, we retrospective analysis the clinical, pathological, treatment and follow-up treatment of 15 cases of FM. The expression of CD103 in all cases was detected by immunohistochemistry.

**Result:**

A total of 15 patients were enrolled, 7 were primary follicular mucinosis (P-FM) and 8 were mycosis fungoides-associated follicular mucinosis (MF-FM). Lesions of both P-FM and MF-FM are difficult to distinguish, present with red or dark red plaques and follicular papules. Pathologically, MF-FM showed more significant infiltrates of folliculotropic lymphoid cells, and the amount and proportion of CD103+ cells were significantly higher than that in P-FM. Follow-up data were available for 13 patients. Three cases were resolved after surgical resection, two patients were marked improved after oral administration of hydroxychloroquine and three times ALA photodynamic therapy respectively. The rest patients showed only modest efficacy.

**Conclusion:**

FM should be differentiated based on pathological characteristics and treatment response, CD103 is helpful in differential diagnosis of FM.

## Graphical Abstract

Comparison in ROC space. 
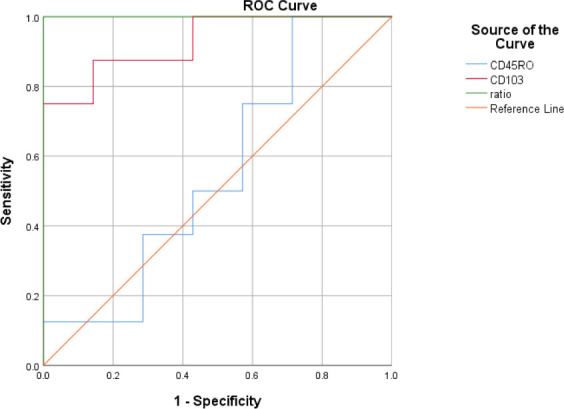

## Introduction

Follicular mucinosis (FM), also known as Alopecia mucinosa, is an epithelial reaction pattern characterized by mucin deposition in the outer root sheath and sebaceous gland. The pathogenesis of FM is not completely understood. There are some controversies in FM include nomenclature, biologic behavior, and treatment options. Firstly, it is not suitable to be named alopecia mucinosa, since most cases do not occur on the scalp. Secondly, some regard FM as an inflammatory process but with a tendency for clonal lymphocyte proliferation, but Ackerman proposed that FM was a form of cutaneous T-cell lymphoma (1). Therefore, it is of great clinical significance to distinguish the nature of FM. Finally, treatment options for FM are very difficult, and actually most treatment options are not effective in most cases. CD103 is the surface marker of Tissue resident memory T cell (TRM cell). MF is considered to be a TRM cell tumor (2). In this study, 15 cases of FM were collected to analyze the clinicopathological features and to explore the application value of CD103 expression in determining the nature of FM.

## Materials and methods

All patients were collected in the dermatology department of the Affiliated 3rd Hospital of Hangzhou from 2014 to 2021. Diagnosis of FM was mainly depended on pathological findings, clinical follow-up and treatment effect, and other possible diseases are excluded. Ematoxylin and eosine stained tissue sections were analyzed. Immunohistochemical studies were performed with antibodies against CD3, CD4, CD5, CD7, CD8, CD20, CD30, C45RO, CD56, TIA1 cytotoxic granule associated RNA binding protein, and Ki67. The expression of CD103 (Abcam, ab224202, 1:100 dilution) in all cases was detected by immunohistochemistry. Appropriate positive and negative controls were included for all antibodies tested. Immunohistochemical positive cells of 5 non-overlapping high-power fields of follicle (×400) were counted by Image Pro Plus 6.0 software. The ratio of CD103/CD45 in follicle was calculated, and the data were statistically processed by SPSS25.0 software. The Medical Ethics Committee of Hangzhou Third hospital, reviewed and approved the protocol (2021KA001), and all patients provided written informed consent.

## Results

### Clinical features

The patients included four children (age <18 years) and 11 adults (age ≥18 years), ranged from 9 to 86 years (mean age 33.8 year; median age 32 years). The duration of clinical symptoms before diagnosis ranged from 1 week to 10 years. Eleven cases were limited to the face and neck, and four cases involved the face, limbs and trunk simultaneously. The lesions may present as red or dark red plaques, follicular papules with irregular shape and infiltration, with a small amount of scales on the erythema in two patients. Four cases involved the scalp with alopecia. There was no atrophy in the skin lesions. One patient had mild pain, two patients had mild itching, and the rest had no obvious subjective symptoms. Among the 15 patients, 7 were primary follicular mucinosis (P-FM) (6 localized, 1 generalized) and 8 were mycosis fungoides-associated follicular mucinosis (MF-FM) (6 localized, 2 generalized). Routine laboratory examinations such as blood routine, urine routine, hepatic and renal function, and autoantibody series were within normal limits.

### Histopathology

Most of the patients had mild to moderate hyperkeratosis and parakeratosis. Reticular degeneration of epithelial cells in the outer root sheath and sebaceous gland was observed with cystic fissure and mucin accumulation. Alcian blue staining was positive in all patients. There were different degree of lymphocytes infiltration in follicular epithelium, and eight cases of MF-FM showed prominent lymphocyte atypia and epidermotropism. All the 15 cases had dense or scattered eosinophils infiltrates.

### Immunohistochemistry

All the cases showed CD3(+), CD4(+), CD5(+), CD20(−), CD79a(−), CD8(+)(3/15), CD30(+−)(1/15), TIA-1(+−)(2/15), granzyme B(+−)(1/15). Table 1 illustrates the result of CD45RO count, CD103 count, and CD103/CD45RO ratio in both the follicle compartments of P-FM and MF-FM groups. There was no significant difference in CD45RO positive cells between the two groups (*p* = 0.74), while the ratio of CD103 and CD103/CD45RO in MF-FM group was significantly higher than that in PFM group (*p* = 0.01, *p* < 0.01, respectively).

**TABLE 1:** CD45RO, CD103, and CD103/CD45RO ratio in follicle compartments of P-FM and MF-FM.

	P-FM (*n* = 7)	MF-FM (*n* = 8)	*p*
CD45RO/5HPF	56.71 ± 36.07	62.50 ± 30.06	0.74
CD103/5HPF	10.43 ± 8.40	38.38 ± 15.92	0.01
CD103/CD45RO	0.22 ± 0.10	0.64 ± 0.15	<0.01

### Treatment and follow-up

**FIGURE 1:**
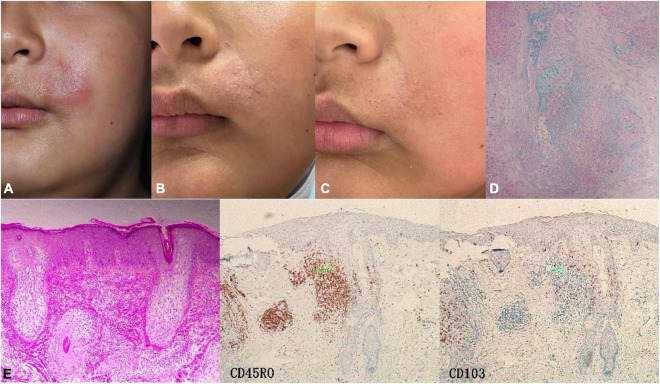
Case3 (P-FM). **(A)** The boy presented with perioral infiltrating red plaque on the left side at first visit. **(B)** After 2 months of oral hydroxychloroquine, most of the lesions subsided with a little scale. **(C)** After 5 months of oral hydroxychloroquine, the skin lesions basically regressed, leaving enlarged pores. **(D)** Alcian blue staining was positive. **(E)** Histopathology showed enlarged, distorted hair follicle expanded by pools of intrafollicular mucin deposition. IHC, CD45RO (++) CD103 (+–).

**FIGURE 2:**
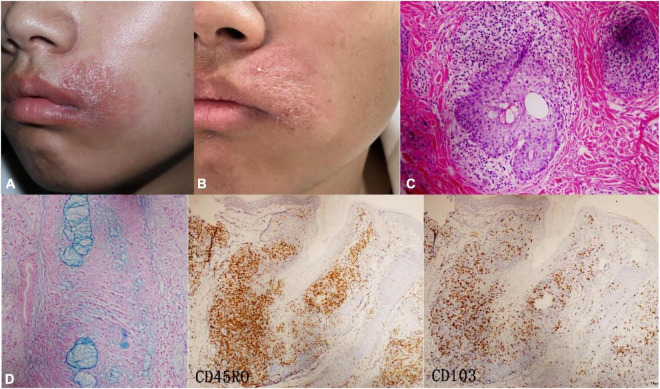
Case4 (localized MF-FM). **(A)** At first visit, the boy presented with perioral red plaque on the left side. **(B)** After three times ALA-PDT, the sense of infiltration significantly decreased. **(C)** Alcian blue staining was positive. **(D)** Histopathology showed a dense infiltration of lymphocytes around the follicle. IHC, CD45RO (+++) CD103 (++).

Follow-up data were available for 13 patients (6–42 months, mean 24.3 months), 6 patients were P-FM, and 7 patients were MF-FM. Of the six P-FM patients, two patients were cured after surgical resection, and one patient was improved after oral administration of hydroxychloroquine (Figure 1). Of the seven MF-FM patients, one patient was cured after surgical resection, one patient was improved by ALA-PDT (Figure 2). The rest patients often had two or more treatments. The main treatment including one systematic glucocorticoids, two NB-UVB phototherapy, three oral hydroxychloroquine, one oral thalidomide and one topical glucocorticoids all showed ineffective.

## Discussion

Follicular mucinosis is a histologic pattern present in a multitude of benign, inflammatory, and neoplastic skin conditions. The clinical presentation of FM is variable, but the typical presentation is dark eczema-like plaques, follicular papules, with or without alopecia and pruritus. The most common lesions occur on the scalp, face and neck. Pathologically, different degrees of mucin deposition and lymphocyte infiltration were observed in the outer root sheath and sebaceous gland (1).

Since Hermann Pinkus first reported six cases of alopecia with mucin deposition in follicular in 1957, which now known as FM, there have been many controversy on the definition, classification, and pathogenesis of FM. The most accepted classification lists a primary form (PFM), which is benign and idiopathic mainly occurring in children and young adults, and a secondary form, typically affecting elderly patients, most commonly associated with MF. The chances of lymphoma appearing in follicular mucinosis patients vary greatly, ranging from 14 to 32% (3). In our study, it even reached more than half, which may be related to different evaluation criteria.

There is no uniform standard for the diagnosis and differentiation of P-FM and MF-FM, and the clinicopathological features of both of them overlap to some extent (4). It is not reliable to judge FM only according to age, lesion morphology and location. MF-FM confined in the head or face of adolescents is not uncommon in literature (5). Among the eight MF-FM collected in this study, six patients had lesions confined to the head and face, and two patients were younger than 18 years old.

Pathologically, Mehregan (6) noted that the dominant infiltration of eosinophils suggested primary benign FM, but different amounts of eosinophils could be observed in all our cases, and there were a considerable number of eosinophils in some MF-FM lesions. Monoclonal studies of T cell rearrangements have also failed to reliably distinguish between FM and MF. In a study with an average follow-up period of 10 years, TCR rearrangements were clonable in 5 out of 7 PFM patients, but none progressed to MF (5). The difficulty in the diagnosis of cutaneous lymphoma is well illustrated in FM, when a small percentage of neoplastic T cells is present among a larger population of reactive lymphocytes and other inflammatory cells.So, we try to find a molecular marker that could distinguish the properties of FM:CD103 staining was performed on all FM patients. The results showed that both localized MF-FM and generalized MF-FM presented more positive cells than PFM. This is easily explained. MF is considered a TRM cell tumor (7), and tumor cells often express the TRM cell markers CD103 and CD69 (2). CD103 interacts with the E-cadherin expressed by keratinocytes and is essential for TRM cell residency in the epithelium (8). CD103 specifically marked the folliculotropic/epidermotropic lymphocytes that resided in the epidermis for a long time, and the number of CD103-positive lymphocytes infiltrated by MF-FM was higher than that of PFM under the background of CD45RO with similar infiltration degree. Therefore, we believe that CD103 is expected to be the basis of classification and diagnosis of FM. In addition, in a study of 203 cases of Folliculotropic Mycosis Fungoides, the morphology of the lesion and the degree of perifollicular lymphocyte infiltrates were also considered to be the basis for distinguishing indolent Folliculotropic Mycosis Fungoides with an aggressive form (9). Due to the limitation of sample size, we expect to confirm the results in a larger sample study. There may be more precise and scientific technical methods and judgment basis to distinguish the two subtypes in the future.

The course of FM is chronic, and spontaneous remission is rarely reported. None of the treatments has been shown to be consistently effective. Local resection may be the best choice for the treatment of localized FM. In this study, three patients with limited skin lesions had no recurrence after cosmetic surgery, so it is recommended to remove the lesions as completely as possible during pathological examination. The treatment of MF-FM should refer to classical MF, including topical drugs, phototherapy and various systemic drugs. However, like folliculotropic MF, NB-UVB and other methods commonly used to treat MF are often less effective due to the special location and depth of lymphocyte infiltration (10). In the case of poor response to phototherapy and oral medication, local radiotherapy or local ALA-PDT therapy is recommended for localized MF-FM, while systemic chemotherapy is recommended for generalized MF-FM. The treatment of MF-FM has a slow onset, difficult remission and poor prognosis. Therefore, early diagnosis, treatment and long-term follow-up of FM are very important.

In summary, we described the clinicopathological features and CD103 expression in 15 FM patients. The nature of FM is still unclear, and it is difficult to distinguish PFM with MF-FM based on its clinical, pathological and molecular results, but their biological behavior and therapies are different. The expression of CD103 is expected to play a useful role in the determination of FM properties.

## Data availability statement

The original contributions presented in this study are included in the article/Supplementary material, further inquiries can be directed to the corresponding author.

## Ethics statement

The studies involving human participants were reviewed and approved by the Medical Ethics Committee of Hangzhou Third hospital (2021KA001). Written informed consent to participate in this study was provided by the participants’ legal guardian/next of kin. Written informed consent was obtained from the individual(s), and minor(s)’ legal guardian/next of kin, for the publication of any potentially identifiable images or data included in this article.

## Author contributions

PW, MZ, and HS contributed to conception and design of the study. YW, LL, and HZ collected the data. JW wrote the first draft of the manuscript. All authors contributed to manuscript revision, read, and approved the submitted version.
