# Supplementary material for: Clinicopathological features, therapeutic options, and significance of CD103 expression in 15 patients with follicular mucinosis
Source: Front Med (Lausanne). 2023 Feb 17;10:1032072. doi: 10.3389/fmed.2023.1032072 (PMC9981639; doi:10.3389/fmed.2023.1032072)
Supplement: Supplementary file 1 [file Table_1.pdf]

Supplementary table 1： Clinical data of 15 patients

	Sex and Age	Course	Lesion	Diagnose	Treatment	Follow-up
1	46F	1year	Dark erythema on the forehead	P-FM	Local resection	3years，cured
2	12M	3years	exudative erythema on right temporal and left cheek	Localized MF-FM	Topical glucocorticoid/tretinoin、betacarotene、systemic glucocorticoids	3years, no improvement
3	9M	2months	perioral infiltrating red plaque on the left side	P-FM	hydroxychloroquine	6months, improved
4	14M	2months	perioral erythema on the left side	Localized MF-FM	NB-UVB、systemic glucocorticoids、acitretin、ALA-PDT	13months, improved
5	51F	2years	Painful erythema on face, trunk and limbs, alopecia	Generalized MF-FM	thalidomide、Tripterysium、diosmin	3years+，no improvement
6	22M	2years	erythema on face, trunk and limbs	P-FM	hydroxychloroquine	2years+，no improvement
7	15F	3years	Red papules on left canthus	P-FM	Local resection	2years+，cured
8	32F	10years	Erythematous and papules on left eyebrow, alopecia	localizedMF-FM	laser、hydroxychloroquine	3years，no improvement
9	23F	2years+	erythema on the forehead	localizedMF-FM	Local resection	3years，cured
10	39F	1week	erythema on face, trunk and limbs, alopecia	P-FM	systemic glucocorticoids、hydroxychloroquine、thalidomide	12months，no improvement
11	40F	5months	infiltrating red plaque on the left cheek	P-FM	hydroxychloroquine	3years，no improvement
12	27M	6months	Erythema on the jaw	P-FM	unclear	lost
13	37M	2weeks	Papule on the jaw and neck	localizedMF-FM	acitretin	12months，no improvement
14	54M	3years	follicular papule on the face,trunk and limbs,alopecia	Generalized MF-FM	NB-UVB	12months，no improvement
15	86F	2years	erythema on the forehead	LocalizedMF-FM	unclear	lost
